# Quality improvement collaborative to increase access to caesarean sections: lessons from Bihar, India

**DOI:** 10.1136/bmjqs-2024-017454

**Published:** 2025-02-20

**Authors:** Abha Mehndiratta, Prabir Ranjan Moharana, Tanmay Mahapatra, Sridhar Srikantiah, Sunil Babu, Sarita Simba, Sanjiv Daulatrao, Vikas Pandey, Rahul Shastri, Srinivas Kodiyath, Sulagna Mukherjee, Pramod Sah, Pierre Barker

**Affiliations:** 1 Institute for Healthcare Improvement, New Delhi, India; 2 Care India, New Delhi, Delhi, India; 3 State Health Society, Bihar, India; 4 Global Health, Institute for Healthcare Improvement, Cambridge, Massachusetts, USA

**Keywords:** Cesarean delivery, Collaborative, breakthrough groups, Healthcare quality improvement, Obstetrics and gynecology, Quality improvement

## Abstract

**Background:**

Countries with resource-poor health systems have struggled to improve access to and the quality of caesarean section (C-section; CS) for women seeking care in public health facilities. Access to C-section in Bihar State remains very low, while access has increased in many other contexts.

**Methods:**

We used quality improvement (QI) combined with targeted resource management to test and implement changes that were designed to increase C-section delivery. We compared C-section delivery percentages after the interventions across eight intervened (QI) hospitals and between QI hospitals and the remaining 22 non-intervened (non-QI) hospitals with baseline CS <10%. We linked patterns of improvement and sustainability to theoretical drivers of improvement and timing of interventions.

**Results:**

In QI hospitals, C-section percentage increased from 2.9% at baseline to 5.9% in the intervention phase and 4.6% in the post intervention phase. In non-QI hospitals, we observed a small change (2.6–3.3%) during the same time period of the interventions in the QI hospitals. Addition of skilled personnel resulted in increased C-section percentage in QI hospitals (3.6–5.9%) but not non-QI hospitals (3.4–3.2%).

**Conclusions:**

C-section availability increased for a population of women giving birth following initiation of QI BTS collaborative in a low-income country public sector setting that has historically struggled to provide this service. Addition of obstetric and operating room resources alone, without interventions to support system changes, may not result in additional increase in C-section delivery. The adaptive implementation model may contribute to efforts to provide more access to C-sections in other very resource-limited settings.

**WHAT IS ALREADY KNOWN ON THIS TOPIC**

- Across the globe, the proportion of women delivering by caesarean section (C-section) has increased since 1990, but has remained persistently low in low-income countries while soaring rapidly in middle-income countries.

**WHAT THIS STUDY ADDS**

- This study increased access to C-sections for women in a poor resource setting like Bihar state, and it shows the importance of quality improvement (QI) to create the enabling environment for other changes (eg, addition of trained personnel) to take hold.
- QI plus resources (vs QI alone or resources alone) are required interventions to effect change.

**HOW THIS STUDY MIGHT AFFECT RESEARCH, PRACTICE OR POLICY**

- This initiative demonstrated that a context-sensitive collaborative approach for QI, in collaboration with local government, is an effective method to increase C-sections in poor economies.

## Introduction

 Caesarean section (C-section) is a surgical intervention for childbirth, which, if conducted when indicated, can be lifesaving for both the mother and the unborn baby.^1^ The procedure has also generated significant concern regarding both its non-availability and overuse, as well as its safety.^2^ While the global C-section percentage has increased from around 7% in 1990 to 21% in 2019,^3^ the proportion of women receiving C-section has remained persistently low in low-income countries and has soared rapidly in middle-income countries.^4^ While the truly optimal percent of C-section for a subpopulation of women varies, data published by the WHO suggest no overall population level benefit for maternal survival with occurrence above 10%.^1^ The skewed distribution of C-section deliveries by country, wealth and facility type is demonstrated within low-and-middle-income countries (LMICs) where large within-country variations in C-section percentage are seen.^5^

The women of India, like many others in emerging economies with geographically non-uniform development, face challenges in accessing safe and effective childbirth. Depending on geography, socioeconomic status or available health facility infrastructure, women may struggle to have seamless and rapid access to a skilled C-section delivery option when needed.^6^ They may also be routinely offered C-section when natural delivery is the appropriate means of childbirth for the mother and new born,^7^ resulting in unnecessary surgical deliveries. Women in LMICs who are able to access C-sections often face major safety risks; African women undergoing C-section are 50 times at risk of death compared with women in high-income countries.^8^

We undertook a quality improvement (QI) initiative in the Indian State of Bihar to increase the provision of C-section in a third of the state’s district hospitals, the secondary care component of the public healthcare system in Bihar. Bihar is a resource-poor, highly populated (third highest in the country) Indian state that reports variations in C-section percent by facility type.^6^ While the proportion of C-section deliveries are slowly increasing in Bihar (from 2.3% (2015–2016) to 5.3% (2020–2021)),^9^ access to C-section remains significantly low (3%) in public facilities, with C-section availability threefold lower than in private facilities (10.3%).^10^

While many efforts are underway to curtail the excessive use of C-section in emerging and high-income economies,^11^ driven by WHO recommendations,^12^ the difficult task of increasing access and improving quality of C-section for women seeking delivery care in public healthcare facilities in resource-poor countries has not received similar attention. QI implementation strategies have been used successfully to increase the uptake of vaginal delivery for low-risk women^11^ and to decrease C-section deliveries in middle- and high-income countries.^13^ We used a similar approach—a QI collaborative design^14^—to test and implement a set of change ideas to increase C-section deliveries in 10 of the 36 District Hospitals in the State. The aim of the initiative was to increase the C-section percentage to above 10% in the participating hospitals over a 16-month period of implementation. We quantitatively assessed the impact of the collaborative on our improvement aim and report on our experiences of implementation, including a qualitative assessment of the barriers to and enablers of change

## Methods

The efforts to improve access to C-section were undertaken in Bihar State, India, which has maternal and neonatal mortality rates well above national averages.^15 16^ The improvement initiative was supported by the Government of Bihar (GoB) and development partners (Institute for Healthcare Improvement (IHI) and CARE India).

The QI initiative was undertaken in 10 district hospitals chosen by the State Health Society of Bihar, 5 of which had been involved in a previous QI collaborative. The goal of the previous QI collaborative was to increase accurate and transparent reporting of maternal and newborn complications and deaths. One of the hospitals used QI to successfully increase C-section delivery percentage from baseline median of 8% to 14%. There was a 6-month interval between the end of the previous project to the start of the new project. While we provided guidance to include only district hospitals with C-section percentages <10% in the QI initiative, the project was asked to include two hospitals with a proportion of C-section delivery >10% at the start of the initiative. The GoB provided essential resources for maternal and newborn care to the district hospitals. Medical staff from participating hospitals were all recruited and paid for by the Government of Bihar and were not financially incentivised to undertake this work. The provision of services for the population was entirely funded by the State of Bihar, and no insurance schemes were active for this population at the time. The Government of Bihar also provided comparative data on percentage of C-section deliveries for the remaining 26 district hospitals that were not included in the QI collaborative.

District hospitals in Bihar deliver an average of around 600–800 women each month, with seasonal increases to around 1000–1200 births per month. While district hospitals have obstetric-trained medical and nursing staff, and government mandated clinical protocols and associated clinical trainings to improve care pathways for women receiving obstetric care, site-based surveys, conducted at a midway point during the intervention,^17^ showed variation in labour room and operating theatre infrastructure availability, available human resources, supply of consumables, reported patient satisfaction and frequency of QI meetings across 10 participating QI hospitals (online supplemental table 1).

### The intervention

Ten district hospitals in Bihar State were convened to create a learning network to identify, test and document innovative ideas to improve the quality of maternal and newborn care. The improvement initiative was implemented with an IHI Breakthrough Series (BTS) Collaborative Design.^14^ While the initiative had broader clinical goals to improve the quality of maternal and newborn care, we focus here on efforts to improve access to C-section services. The intervention used existing clinical protocols and standards for maternal and newborn care and aligned with existing government quality programmes, the LaQshya programme for QI in labour rooms and operating theatres,^17^ and the National Quality Assurance Standards.^18^

The intervention in the QI hospitals began with an orientation of health leaders from the state (Executive Director of State Health Society and State Programme Officers for Quality and Maternal Health) and 10 participating districts (District Programme Managers and Civil Surgeons) in February 2018. QI training was provided through two workshops of 3 days from February to April 2018. The BTS included three learning sessions, spaced 4 months apart, and monthly visits by QI coaches provided by CARE India and IHI. A 2-day clinical training session was held in May 2019 on safety of vaginal deliveries, C-sections and blood transfusions. IHI provided technical support for QI design and methods, and built the technical capability of GoB leaders in leading and managing QI. Both IHI and CARE India trained key partners and leaders in QI methods.

Each participating QI hospital formed a QI team consisting of a champion doctor, champion nurse, hospital manager, medical superintendent and improvement coaches who participated in learning sessions and undertook local improvement. Monthly leadership updates were shared by the district hospital to the Civil Surgeon’s office, which reports to the District Magistrate. The Civil Surgeon offices held district maternal death reviews and monthly QI meetings to review maternal and newborn processes outcomes, but their frequency and quality varied. Regular updates on the QI Collaborative were shared with the State Health Leadership. The collaborative concluded in May 2019, followed by a 9-month observation period. Observations ceased in March 2020 due to the onset of the COVID pandemic.

Both QI and non-QI hospitals received the same technical support from CARE India directed at optimising existing obstetric resources, improving operating theatre services, and the identification and management of maternal complications. A state-wide convening of all hospital leaders (QI and non-QI hospitals) was facilitated by Bihar state health leadership in early December 2018 to discuss the importance of providing access to C-section for eligible women, and a state-wide recruitment drive November 2018–January 2019 provided supplemental obstetric trained staff and anaesthetists to support C-sections. The resulting additional obstetric and operating room staff were allocated equally to QI and non-QI hospitals.

### Project theory

**Figure 1:**
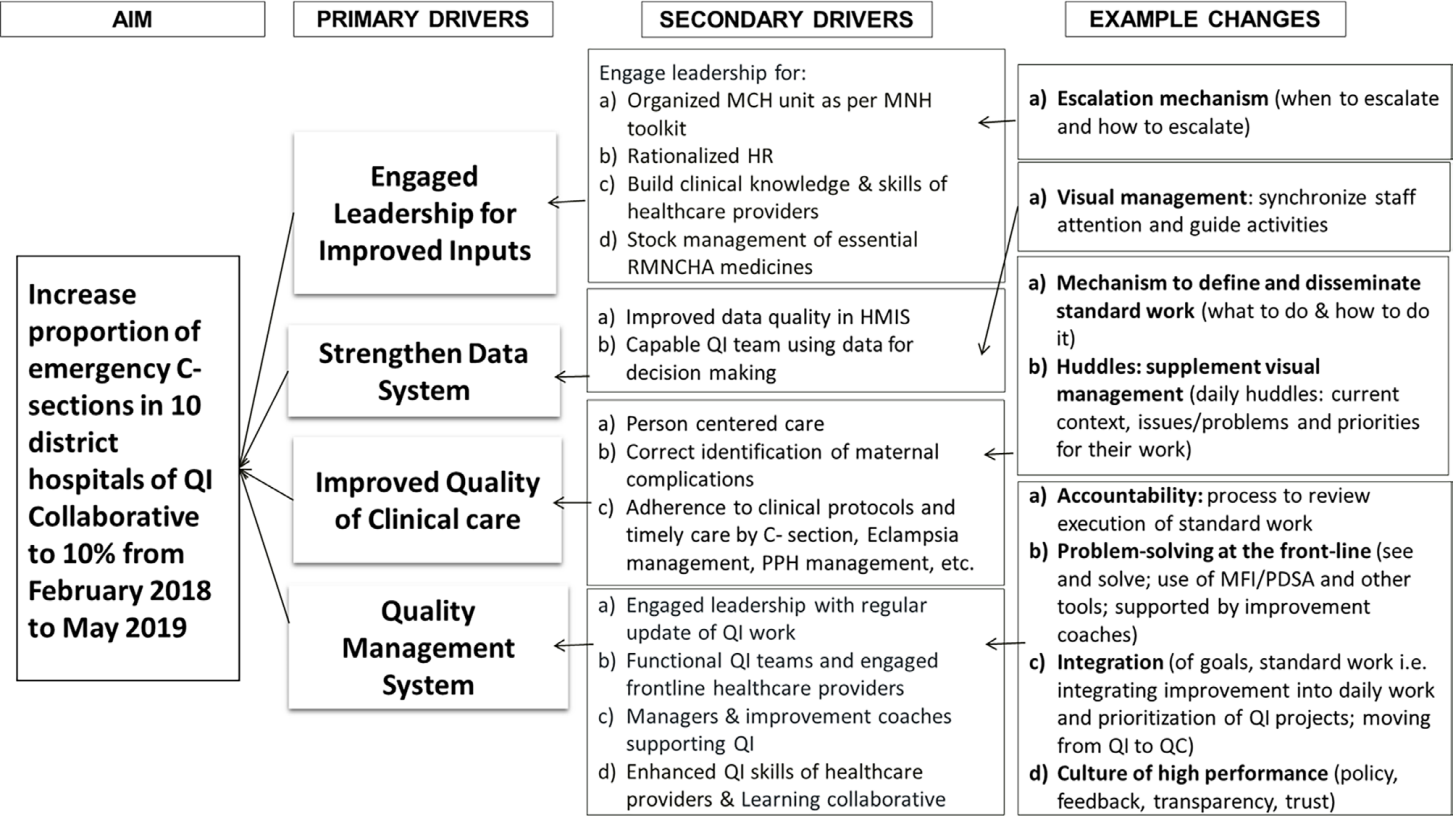
Driver diagram showing collaborative aim, four primary drivers, linked secondary drivers and examples of changes. The primary and secondary drivers were constant for all hospitals for the duration of the collaborative. HMIS, health management information services; HR, human resources; MCH, maternal child health; MFI, model for improvement; MNH, maternal newborn health; RMNCHA, reproductive, maternal, newborn, child health, adolescent; PDSA, plan do study act; PPH, postpartum haemorrhage; QC, quality control; QI, quality improvement; QP, quality planning.

The theory of change emphasises four theoretical drivers, depicted in a ‘Driver Diagram’ in figure 1, that are accepted determinants of improved health system performance^19^: leadership engagement, data system strengthening, clinical care quality improvement and effective quality management. We added a further two drivers that were felt to be key drivers of success—inputs, and patient- and family-centred care—during the course of the initiative These drivers generated specific health service strengthening ideas to improve the quality and availability of C-section delivery option in district hospitals of Bihar. The detailed change ideas associated with these drivers are detailed in online supplemental table 2 and the full change package that includes all the changes tested is detailed in online supplemental table 3.

### Study of the intervention

We used Statistical Process Control^20^ to confirm the stability of C-section occurrences during the pre-intervention period, and monitor changes in C-section occurrences during and after the intervention. The method of ‘freezing’ the mean values of processes and outcomes^21^ was used to detect special cause variation (eg, a shift in average C-section percentage) and to adjust the mean C-section percentage accordingly. The mean C-section percentage was ‘frozen’ at the end of the baseline period until the first special cause was detected. We did not adjust the mean for 1 or 2 values detected outside upper or lower control limits (special cause variation) but reported those values with possible attribution to known interventions or changes in the environment.

Given that the goal of the initiative was to increase C-section delivery percentage to >10%, our analysis excluded two QI hospitals and four non-QI hospitals that had baseline C-section percentages >10%. The analysis compared the C-section percentages of 8 QI hospitals with 22 remaining non-QI district hospitals across Bihar.

### Measures

C-section and total delivery data were collected for the baseline (10 months), intervention (16 months) and sustainability (9 months) periods. The outcome measure was the proportion (%) of institutional deliveries conducted by C-section. Data on all deliveries and C-sections performed were collected from local hospital records and Hospital Management Information System (HMIS) from April 2017 to February 2020. We had complete monthly data for 35 hospitals. One non-QI hospital had missing data for the last 8 months of the observation period.

The district hospital teams worked to create a reliable system for capturing data on deliver mode outcome of all pregnant women admitted in the IPD and Labour Room Register. A daily summary report was filled by the Labour Room staff for local review, visual display and quality improvement. Monthly data were uploaded in the State HMIS. The data for C-sections and total number of institutional deliveries used in this manuscript are obtained from the HMIS.

Qualitative data to assess the relative impact of the change ideas related to the four key drivers were collected on an ongoing basis by the quality coaches from IHI and CARE during their weekly visits, and assembled into a ‘live’ tracking document, managed by the IHI team. The assessment as to the relative impact of the change ideas on the processes of care and on C-section delivery percentage was derived from a subjective assessment of the degree of improvement of care processes and objective assessment of the rate of change of C-section delivery percentage.

### Ethics

The improvement initiative across the 10 hospitals was approved by the GoB. The QI collaborative evaluation study (which also included the HMIS data use to compare QI non-QI hospital) protocol and procedures were reviewed and approved by the Ashirwad Ethics Committee, Ashirwad Hospital and Research Center. We used aggregated, deidentified data generated from HMIS for the purpose of the programme monitoring as part of governmental programme implementation and to compare performance of QI and non-QI hospitals.

The Bihar Continuous Quality Improvement (QI) was a joint effort of the GoB, CISSD and IHI, funded by the Bill and Melinda Gates Foundation.

## Results

The core design, timeline and content of the intervention did not change significantly over time. Leaders and hospital teams participated in scheduled sessions and received support as planned. Important activities and events that might have influenced the results are noted in the figures provided.

**Figure 2:**
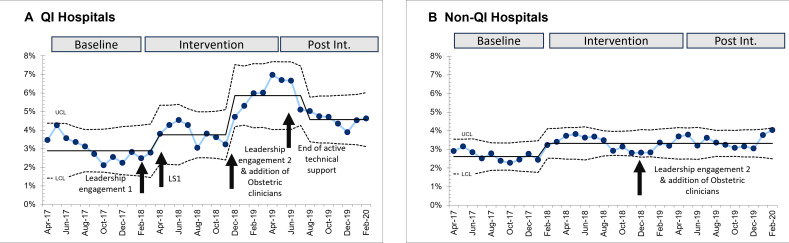
Caesarean section (C-section) percentages in quality improvement (QI) and non-QI hospitals. Equivalent baseline, intervention and post-intervention (post int.) periods for non-QI hospitals are indicated. February 2018: Leaders of the district level health system/hospitals were engaged. April 2018: First learning session (LSI, QI hospitals). December 2018: Catalytic meeting conducted by the project sponsors (Secretary Health/Executive Director State Health Society) with district leaders (civil surgeons, district programme managers) to revive commitment to address ongoing quality issues through engagement in the collaborative (all hospitals). January 2019: State recruitment of doctors—obstetricians, anaesthetists and EmONC trained doctors (all hospitals). June 2019: The collaborative ended, and active support of QI hospitals was discontinued. OB, obstetric.

Hospitals with baseline C-section deliveries <10% included 165 991 deliveries in QI hospitals and 453 296 deliveries in non-QI hospitals. The average monthly deliveries in the QI hospital group (625/month) was similar to non-QI hospitals (600/month) during the same time intervals. C-section percentages in the baseline period were similar in QI (2.9%) and non-CS hospitals (2.6%). We observed a 106% relative increase in CS compared with baseline during the intervention period (2.9–5.9% C-section deliveries) which dropped to a 57% relative increase (2.9–4.6% C-section) in the post-intervention period. The comparative changes in the non-QI hospitals were 26% (2.6–3.3% C-section deliveries) in the corresponding months of the intervention and no further change (3.4% C-section) in the corresponding post-intervention months (figure 2).

Two phases of improvement in C-section delivery percentage were observed in QI hospitals. The first increase happened after initial leadership to publicise the need for more C-section availability in the State, and launch of learning sessions 2 months later (2.9–3.6% C-section). The second increase (3.6–5.9% C-section) followed state-level meetings to reinforce the need to provide C-section services and efforts to recruit obstetricians. The non-QI hospitals saw a similar small increase from 2.6 to 3.3 C-section percentage at the time of the start of the QI initiative with no further increases after state-level meetings to promote C-section services and recruitment of obstetricians (figure 2).

Two hospitals participated in the QI collaborative that had baseline (pre-intervention) C-section percentages >10% are analysed separately. For the remaining eight hospitals, six hospitals showed evidence of increase in C-section percentage compared with baseline. We assessed the impact of the theoretical drivers of improvement on the high C-section hospitals and in three categories of hospitals based on baseline C-section delivery rate and response to the QI initiative

**Figure 3:**
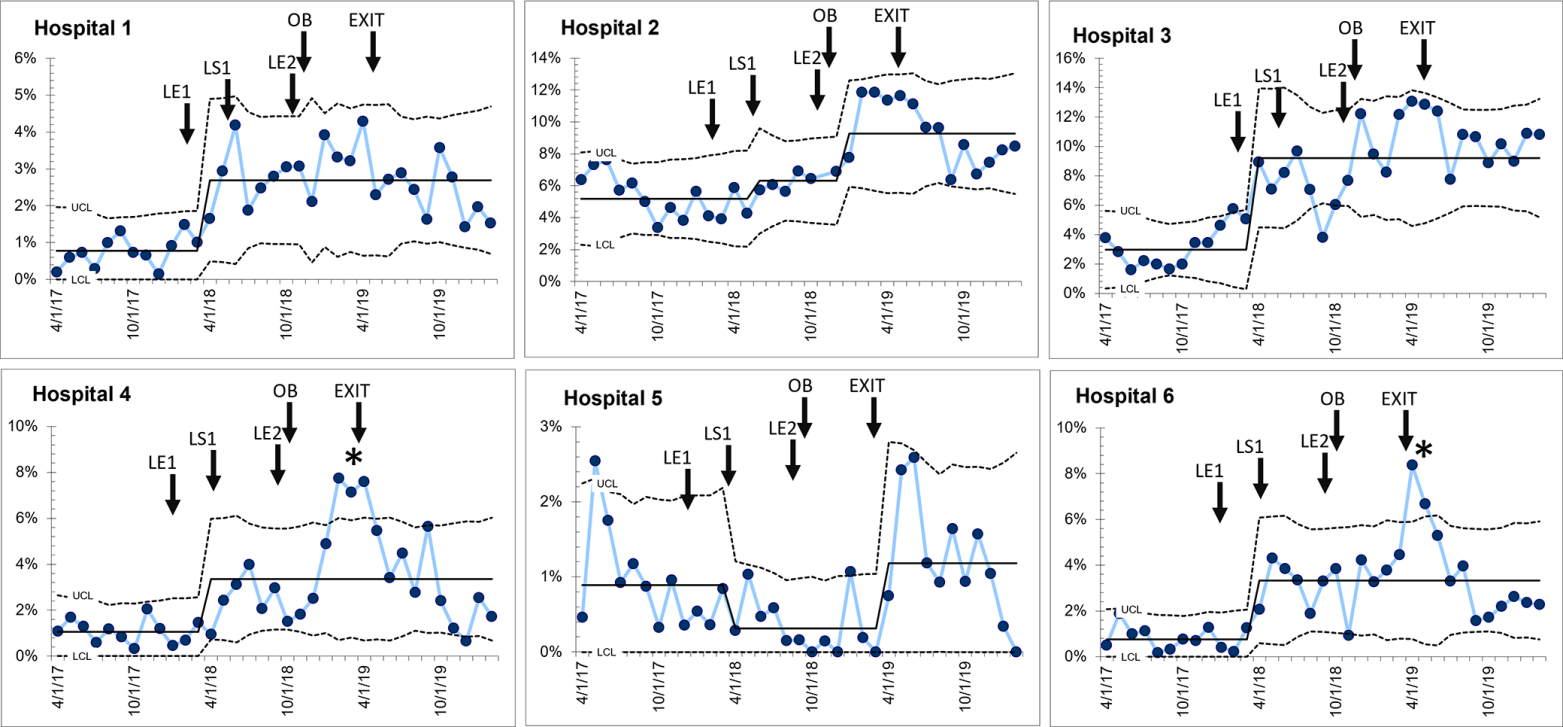
Six hospitals that demonstrated sustained increase in caesarean section (C-section) percentage after initiation of the quality improvement (QI) collaborative. LE1—Leadership engagement meeting 1; LS1—Learning session 1; LE2— Leadership engagement meeting 2; OB—recruitment of obstetric trained personnel; EXIT—Last month of external QI support for hospital teams and leadership.

*Group 1*: Six hospitals with baseline C-section delivery percentage <10% demonstrated an average threefold increase in C-section percentage during the intervention (2.1–6.0%) and in all hospitals we observed some decrease in these gains after QI support was withdrawn (figure 3). However, average CS delivery percentage for the 9 months after withdrawal of support (4.7%) was sustained at greater than twofold the starting value (figure 3). In two hospitals, the increase in percentage started (as determined by SPC analysis) 3–4 months ahead of LS1, potentially responding to the leadership meeting that was convened in February 2017 to explain the importance of the topic, how it was going to be undertaken and the crucial role of leadership. The second leadership meeting in November 2018 together with the addition of obstetric-trained personnel in December 2018 was followed by a second improvement signal in hospital 2. The hospital with the lowest baseline percent of the collaborative in fact showed worsening after the start of the collaborative with increases only seen after the infusion of obstetric resources. Given that these arrangements to bring in one or two obstetricians/trained surgeons were ad-hoc measures to bridge a longstanding gap (eg, general surgeon undertaking C-sections, a single EmONC trained doctor conducting all C-sections, surgeons temporarily hired in one hospital through grant funding), the progress was not sustained. Five of the six hospitals struggled to sustain the gains over the months after withdrawal of active QI support, and attrition of added obstetric trained technical resources.

**Figure 4:**
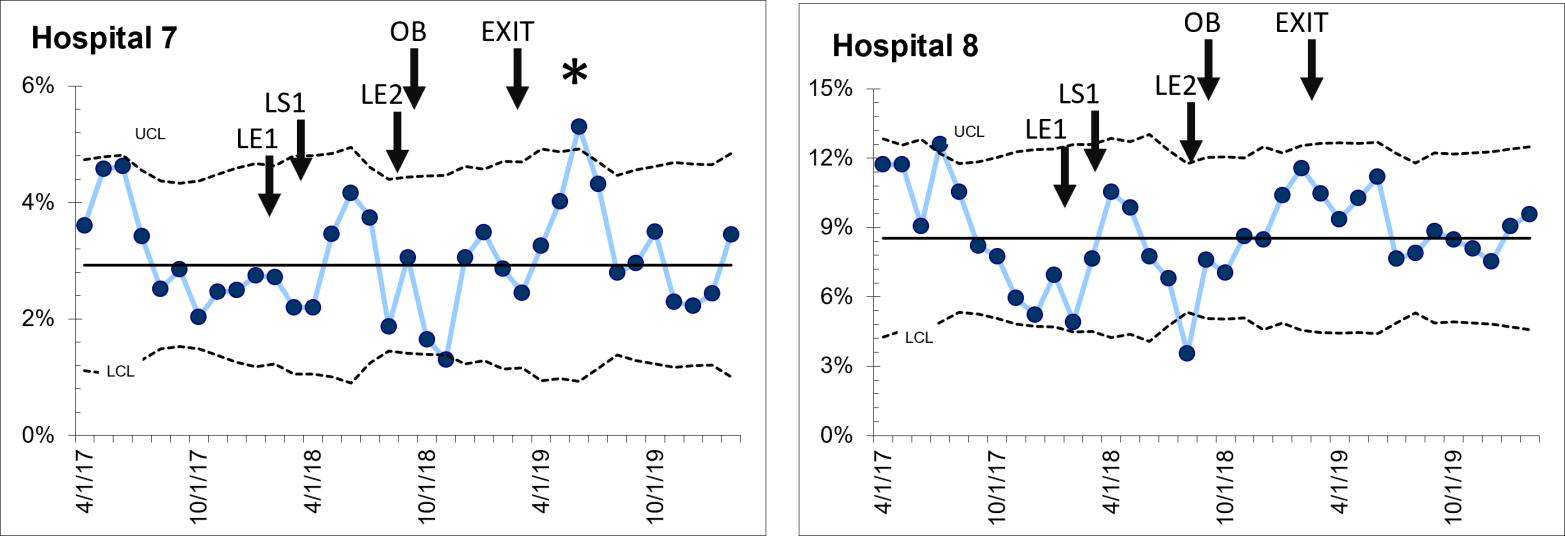
Two hospitals that demonstrated no change in caesarean section (C-section) percentage. LE1—Leadership engagement meeting 1; LS1—Learning session 1; LE2—Leadership engagement meeting 2; OB—Recruitment of obstetric trained personnel; EXIT—Last month of external quality improvement (QI) support for hospital teams and leadership.

*Group 2:* Two hospitals did not show any change in C-section percentage during or after the QI intervention (figure 4), with no shift from baseline after the start of the collaborative, or after the infusion of resources. One hospital had a transient increase in C-section percentage (special cause detected) over the course of 4 months after obstetric staff resources were added. In both hospitals, C-section percentage after the active support for the collaborative was withdrawn was similar to baseline, suggesting that the QI process did not influence the performance of C-sections. These hospitals had very little or no local leadership support to resolve barriers.

**Figure 5:**
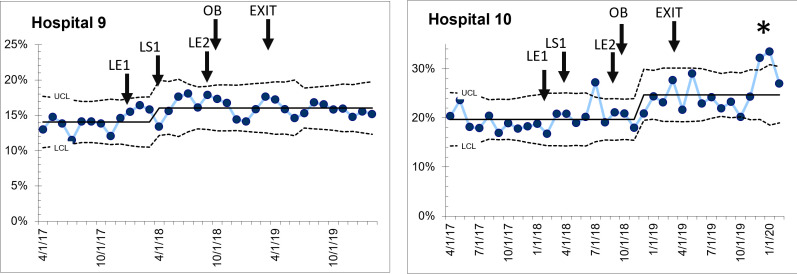
Two hospitals with baseline caesarean section (C-section) percentage >10% at the start of the initiative, that showed further sustained increase in C-section percentage. LE1—Leadership engagement meeting 1; LS1—Learning session 1; LE2—Leadership engagement meeting 2; OB—Recruitment of obstetric-trained personnel; EXIT—Last month of external quality improvement (QI) support for hospital teams and leadership.

*Group 3:* Two hospitals had C-section percentages >10% at the start of the intervention. One hospital had increased its C-section from 6% to 14% during a previous QI collaborative by maximising its capacity (expansion of OT assistants, increased postoperative beds, etc). The hospital had a small increase in absolute C-section percentage (14–16%) shortly after the commencement of the current intervention. The second hospital had stable C-section percentage of 19.7% before the start of the initiative, that increased to 24.7% after infusion of further obstetric-trained personnel (figure 5) with ongoing increase in C-section percentage in the sustainability phase, after withdrawal of QI resources.

*Results in the non-QI hospitals:* In the 22 non-QI hospitals with baseline C-section rates <10%, we observed similar baseline CS delivery percentages as in QI hospitals (2.9% QI, 2.6% CS). In the first 10 months of the intervention, we saw small increases in the CS rate in both QI and non-QI hospitals (to 3.8% QI, and to 3.3% non-QI). The QI and non-QI hospitals responded differently to the added interventions (leadership engagement and additional of obstetric resources): QI hospitals increased C-section delivery percentage to 5.9%, whereas we observed no change in non-QI hospitals (from average of 3.4% to 3.2%) when resources were added.

### Sustainability

After withdrawing active QI support, six of the eight hospitals that showed an increase in C-section delivery percentage showed some decline in peak values that were achieved during the intervention. One hospital continued to improve its C-section delivery percentage, likely due to factors unrelated to our initiative. We stopped tracking C-sections in February 2020 due to the COVID pandemic’s impact on the health system. The pandemic and funding issues also hindered advanced plans to transfer QI technical support to the Government of Bihar, including setting up a QI Collaborating Center and a government-led QI curriculum for coaches.

## Discussion

The observed increase in C-section delivery percentage in the eight QI hospitals with low baseline CS percentage and the relatively small of change in C-section percentage in the equivalent non-QI hospitals during the same time periods showed that a systematic quality improvement approach, coupled with leadership support and additional human resources, can increase the availability of C-section in an environment where C-section is difficult to access and to undertake. Hospitals with a high baseline C-section percentage showed an ongoing increase in C-section percentage, illustrating the risks of moving from undersupply to overdelivery of C-section in LMIC settings. Our confidence in assuming a contributory pathway linking our QI efforts and the change in C-section delivery is supported by the association between the collaborative interventions (initiation, further interventions and cessation) and changes in the time-series data. The residual CS delivery remained 59% higher than baseline values, consistent with previous studies have shown persistence of improved processes and outcomes of the performance following QI interventions.^2 22^ We further linked variation in C-section performance across the system according to differences in four health systems drivers across the 10 hospitals.

While QI collaboratives are widely used in LMICs to improve clinical processes, we are not aware of their application to increase C-section availability, as QI efforts typically aim to reduce C-section overuse.^11 13^ The results of this project are significant, given it was implemented in a challenging environment with historically low C-section rates in the public sector, unlike the private sector where C-section rates have surged. Although exact matching was not possible, we compared C-section percentages in the intervention hospitals to the C-section percentages in the remaining non-intervened district hospitals in the state for analysis.

We assessed the impact of health systems factors (leadership, data management, systems management and clinical care delivery) on changes in C-section percentage. Like in other studies,^22^ effective leadership and engagement of clinical champions was linked to improvement in C-section percentage. The burden of data recording and reporting in LMIC primary healthcare settings is well described. Data reporting improved in quality and quantity when linked to project activities.^23^ The role of infrastructure and resources in determining improvement is complex^24^: in our initiative, an adequate supply of clinicians alone did not predict an increase in C-section percentage. A state-wide initiative to add obstetric and anaesthetic clinicians, coupled with leadership support for C-sections, led to sustained increases in C-section percentages in QI hospitals but no change in non-QI hospitals. This suggests that resources alone, without supporting processes, are insufficient for lasting improvement in C-section availability.

Effective approaches to engaging patients and families in improving maternity care has been demonstrated in similar settings.^25^ Our efforts to support women during labour through counselling and empowering family members to be birth companions was successfully modelled in one district hospital, and efforts to spread this idea to other collaborative QI hospitals showed variable uptake.

This programme provided other opportunities for improving the functionality of health systems by linking to national maternal newborn quality improvement programmes (LaQshya programme^17^ and the WHO Maternal Newborn Network^26^) and engaging State and district administrative leadership (ie, District Magistrates). This initiative also provided a common platform for integration of clinical training and QI learning.

Efforts to transfer the capabilities and knowledge required to sustain and scale the initiative were not fully successful. Plans to build durable and renewable QI capability into the GoB healthcare infrastructure, and to scale up the successful implementation strategies were thwarted by a combination of distraction by external events (COVID pandemic, funding constraint, changes in senior leadership and priorities). These realities point to the fragility of gains of externally driven implementation initiatives, even those designed with a view to sustainability and scale.

### Limitations

While our initiative successfully increased C-section delivery percentage, we did not reach our intended target of 10%. The project did not monitor the reasons for C-sections (eg, Robson Classification^27^) nor did we collect reliable data to evaluate the impact of increased C-section availability on mortality and morbidity. It will be crucial to monitor these risks as C-section shifts inexorably from underuse to overuse.

We had limited control over the selection of QI-intervened hospitals, which included hospitals that previously participation in a QI BTS initiative on maternal health, and two hospitals with C-section percentages that were already greater than the 10% target set for the collaborative. We were informed that the allocation of supplemental obstetric and operating theatre personnel was applied equally to QI and non-QI hospitals, but we have no way of corroborating this information.

Our assessment of role of the theoretical drivers of improvement on the high C-section hospitals and in the three categories, while providing useful qualitative data, is susceptible to bias given the small numbers of hospitals involved, and the lack of a formal quantitative comparison of the effectiveness of each change idea tested.

While the collaborative learning approach is highly generalisable in all settings^28 29^ and the general theory of health system drivers that we used are broadly applicable to change strategies for all LMIC settings,^19 22^ some of the ideas for change were highly contextual for the State of Bihar and for the Indian healthcare system.

## Conclusions

This initiative demonstrated an increase in C-section availability for a population of women around the time of birth, following initiation of a QI BTS Collaborative, in a public sector setting that has historically struggled to provide this service. QI interventions alone, not accompanied by sufficient skilled obstetrics staff had limited effect. By contrast addition of obstetric resources in the absence of an improvement redesign strategy had no impact on C-section delivery percentage. The initiative illustrates the fragility of improvement efforts that depend heavily on the presence of external technical support, and the vulnerability of improvement efforts to personnel/priority change and system shocks like the COVID pandemic. The adaptive combined implementation model that was used in Bihar, interfacing deeply with local contexts, can be applied to increase C-section access in other LMIC settings.

## Supplementary material

10.1136/bmjqs-2024-017454 online supplemental file 1

10.1136/bmjqs-2024-017454 online supplemental file 2

10.1136/bmjqs-2024-017454 online supplemental file 3

## Data Availability

All data relevant to the study are included in the article or uploaded as supplementary information.
