# Supplementary material for: Quality improvement collaborative to increase access to caesarean sections: lessons from Bihar, India
Source: BMJ Qual Saf. 2025 Feb 20;34(6):e017454. doi: 10.1136/bmjqs-2024-017454 (PMC12171458; doi:10.1136/bmjqs-2024-017454)
Supplement: online supplemental file 1 [file bmjqs-34-6-s001.pdf]

Appendix Table 1: Mid-collaborative Assessment of Facilities, November 2018

Hospital Number	% LaQshya Labor Room Score (Self-Assessment)	% LaQshya OT Score (Self-Assessment)	Human Resource<br># EmOC/OG<br># Anaesthetist/ Life Saving Anaesthesia Skills (LSAS)<br># Paediatrician<br># Nurses (LR & OT)	Supply of Consumables	Patient Satisfaction Score on Mera Asapatal Platform (Median Jan-Nov'18)	Regular QI team meetings, Frequency
1	91%	78%	OG: 4<br>Anesthetist/LSAS: 1<br>Paediatrician: 2<br>Nurses (LR & OT): 9	Regular supply	63%	No
2	44%	38%	# EmOC/OG – 03<br>(All are on Deputation basis)<br># Anaesthetist/LSAS- 0<br># Paediatrician- 03<br># Nurses (LR & OT)- 14<br>(None for OT)<br>Additionally<br>1 Gynae, 1 Paediatrician, 2 LSAS trained doctors are posted in DH as per new transfer & posting but not joined till date.	Regular supply	63%	Yes, weekly
3	50%	39%	# EmOC/OG: 3<br># Anesthetist/LSAS: 2<br># Pediatrician: 2<br># Nurses (LR & OT): 11	Regular supply	72%	Yes, weekly
4	82%	66%	# EmOC/OG: 2<br># Anaesthetist/LSAS: 3<br># Paediatrician: 0<br># Nurses (LR & OT): 16	Regular supply	79%	Yes, weekly
5	49%	-	# Surgeon -0<br>#Gyne MO-1 (does not operate)<br>#EmOC- 0<br># Anaesthetist: 1<br># Paediatrician: 1<br># Nurses (LR & OT): 10	Inconsistent Supply	51%	Yes, biweekly
6	54%	40%	# EmOC/OG: 2<br># Anaesthetist/LSAS: 2<br># Paediatrician: 1<br># Nurses (LR & OT): 14	Inconsistent Supply	54%	Yes, weekly
