# Supplementary material for: Quality improvement collaborative to increase access to caesarean sections: lessons from Bihar, India
Source: BMJ Qual Saf. 2025 Feb 20;34(6):e017454. doi: 10.1136/bmjqs-2024-017454 (PMC12171458; doi:10.1136/bmjqs-2024-017454)
Supplement: online supplemental file 2 [file bmjqs-34-6-s002.pdf]

## Supplemental Table 2: Key Drivers and Associated Changes

# 1. Engaged Leadership for Improved Inputs

### 1.1 Organize MCH Unit for Person-Centred Design (as per the updated MCH Toolkit)

| Change Concept | Change Idea |
|---|---|
| Create a healing environment | • Create a clean and welcoming space<br>• Ensure comfort and privacy |
| Streamline patient flow | • Identify steps in the patient care process<br>• Decrease Overcrowding<br>• Maximize ease-of-use for providers |
| Change the culture to support improvement | • Build a culture of trust and transparency<br>• Interact and engage with staff to support person-centred care<br>• Demonstrate by doing |

### 1.2 Optimize Use of Resources

| Change Concepts | Change Ideas |
|---|---|
| Maximize provider and staff capacity and capability | • Build clinical expertise<br>• Build paramedical staff expertise<br>• Clarify roles and responsibilities<br>• Share skilled expertise |
| Manage medicine and equipment | • Create and maintain a stock register<br>• Rationalize procurement |

# 2. Data Systems to Support Improvement

### 2.1 Build Reliable Systems for Data Collection

| Change Concept | Change Idea |
|---|---|
| Standardise data collection | • Complete a daily and monthly summary and review of records |

### 2.2 Strengthen Data Quality

| Change Concept | Change Idea |
|---|---|
| Create transparency in data collection and use | • Track and address data, defects, and results for patient-centred healthcare |

### 2.3 Use Data for Improvement

| Change Concept | Change Idea |
|---|---|
| Use available data appropriately for continuous improvement | • Use data in QI meetings<br>• Incorporate storytelling<br>• Use visual display boards<br>• Use data for decision making |

# 3. Quality Management System

## 3.1 Build Leadership Accountability

| Change Concept | Change Idea |
|---|---|
| Create vision and build will | • Regularly review and follow-up QI work |

## 3.2 Create and Nurture Frontline teams

| Change Concept | Change Idea |
|---|---|
| Change the culture to support improvement | • Acknowledge and support improvement efforts |

## 3.3 Build a Cross-district Learning System

| Change Concept | Change Idea |
|---|---|
| Build QI capability | • Build and support functional QI teams |

# 4. Improve Quality of Clinical Care

## 4.1 Effective Triage of Pregnant Women on Admission

| Change Concept | Change Idea |
|---|---|
| Redesign the triage process | • Ensure resource availability<br>• Implement colour coding triage system<br>• Sensitize frontline staff on common issues |

## 4.2 Increase Identification of Pregnancy-related Complications

| Change Concept | Change Idea |
|---|---|
| Create urgency | • Provide appropriate education on maternal complications through different mechanisms |
| Ensure accurate and reliable monitoring | • Improve quality of documentation<br>• Develop measurement framework to guide improvement |

| | |
|---|---|
| Maintain continuity of necessary supplies | • Ensure timely supply of necessary equipment |

### 4.3 Increase Indicated C-Sections

| Change Concept | Change Idea |
|---|---|
| Increase accountability | • Form a c-section response team<br>• Involve leadership to remove barriers and facilitate changes |
| Redesign process to identify and manage maternal complications resulting in c-section | • Sensitize nurses on indications for c-section<br>• Redesign OT for hassle-free c-section services<br>• Conduct referrals with two-way timely communication between Primary Health Centre (PHC) and DH<br>• Ensure post-operative monitoring compliance |
| Redesign instrument sterilization and procurement process | • Designate area for CSSD<br>• Define roles and responsibilities<br>• Regularly monitor compliance<br>• Standardise procurement of required instruments |
| Increase availability of key services and resources | • Increase doctor availability for c-sections<br>• Ensure emergency laboratory tests are available 24/7 |
| Redesign instrument sterilization and procurement process | • Designate area for CSSD<br>• Define roles and responsibilities<br>• Regularly monitor compliance<br>• Standardise procurement of required instruments |

### 4.4. Increase Blood Transfusion for Pregnancy Complications (if indicated)

| Change Concept | Change Idea |
|---|---|
| Redesign the blood transfusion acquisition process | • Communicate need for blood in a timely way<br>• Leadership authorizes emergency cases<br>• Use data collection, monitoring, and dissemination<br>• Increase availability of blood |

### 4.5 Increase Referrals from Primary Health Centres to District Hospitals

| Change Concept | Change Idea |
|---|---|
| Identify and refer cases in timely manner | • Provide training to staff on clinical knowledge and improvement skills<br>• Establish reliable communication across clinical teams for refereed patient<br>• Improve data collection and reporting |
