# Supplementary material for: Quality improvement collaborative to increase access to caesarean sections: lessons from Bihar, India
Source: BMJ Qual Saf. 2025 Feb 20;34(6):e017454. doi: 10.1136/bmjqs-2024-017454 (PMC12171458; doi:10.1136/bmjqs-2024-017454)
Supplement: online supplemental file 3 [file bmjqs-34-6-s003.pdf]

Appendix 1 Supplemental Table 3

Change Package

The Change package lists the primary and secondary drivers and describes the change concepts and specific change Ideas tested by the different teams.

Please note that primary drivers of *Engaged Leadership for Improved Inputs, Strengthen Data Systems*, and *Quality Management System* all focus on systems and processes that, when improved, also enable improvements in the primary driver of *Improve Quality of Clinical Care.*

1. Engaged Leadership for Improved Inputs

1.1 Organize MCH Unit for Person-Centred Design (as per the updated MCH Toolkit)

Change Concept	Change Idea	Changes Implemented
Create a healing environment	Create a clean and welcoming space	• Used wallpaper depicting natural elements (flowers, etc.).<br>• Placed plants outside washrooms and in the corridors.<br>• Installed audio system to play soothing music.<br>• Provided adequate equipment and training for cleaning staff.<br>• Used TV and Public Address System in maternity unit for health education and communication with patients' family and attendants.
	Ensure comfort and privacy	Ante-natal wards:<br>• Provided beds, reclining chairs, and space to walk for pregnant women.<br>• Provided a separate chair for birth companion.<br>Delivery Rooms:<br>• Ensured privacy and dignity via curtains between patients.
Streamline patient flow	Identify steps in the patient care process	• Used dedicated space for registration, triage, antenatal, delivery, and postnatal care.<br>• Reorganized space and completed construction as needed using existing financial allocations.
	Decrease Overcrowding	• Closed extra entry and exit points.<br>• Used a pass system for visitors (one visitor pass per patient).<br>• Restricted flow of visitors (e.g., adequate guards and/or paramedical works placed at registration).<br>• Counselled patients and family member on visiting policy at time

		of admission.
	Maximize ease-of-use for providers	• Re-organized triage and delivery space (e.g., storage of medicines, equipment, files etc.) to provide ease to healthcare providers.
Change the culture to support improvement	Build a culture of trust and transparency	• Labour room (LR) nurses were encouraged by hospital leadership to report complications, left against medical advice (LAMA), abscond, referrals, and near misses without fear of punitive action.
	Interact and engage with staff to support person-centred care	• Civil Surgeons, Chief Medical Officers, Medical Superintendents, Deputy Superintendent (DS), District Program Managers, and Hospital Managers regularly interacted with staff and reviewed progress in improvement work. They encouraged staff to focus on achievement of improvement aims, objectives, and results.<br>• Leadership allowed hospital staff to test infrastructure redesign change ideas to make the hospital more user friendly for both pregnant women and hospital staff. Quality Circle of Labour Room took up SMART aims for improvement in clinical services.
	Demonstrate by doing	• Hospital leaders in one of the district hospitals started doing emergency caesarean section himself, after completion of his training in Comprehensive Emergency Obstetric and Newborn Care (CEmONC) - demonstrating commitment between *word and deed.*

1.2 Optimize Use of Resources

Change Concepts	Change Ideas	Changes Implemented
Maximize provider and staff capacity and capability	Build clinical expertise	• Provided on-going training to all clinical staff via AMANAT Trainings (Mobile Nurse Mentoring Program).<br>• Nurses trained to assist doctors during caesarean section in absence of operation theatre (OT) assistant.
	Build paramedical staff expertise	• MAMTAs (paramedical workers) were trained to support antenatal and postnatal counselling on breast feeding, nutrition, crowd management, etc.<br>• Grade-IV staff trained to sterilize instruments and equipment by autoclaving.
	Clarify roles and responsibilities	• Duty rosters for nurses, doctors, MAMTAs, laboratory technicians, and housekeepers prepared and publicly displayed for transparent and timely rotation of duty.<br>• Roles and responsibilities for all staff clearly stated and communicated.<br>• Dedicated/assigned staff for centralized autoclave responsibilities.<br>• *See Additional ideas for more details on staff roles*.
	Share skilled expertise	• Surgeons and anaesthetists from sub-district hospitals and other CEmONC centres covered c-section services as needed across locations.
Manage medicine and equipment	Create and maintain a stock register	• Labour room "stock outs" decreased by starting a new indenting process to proactively identify needed supplies.<br>• Reduced out of pocket expenditure by properly stocking drugs and supplies in labour room and decreasing unnecessary referrals.
	Rationalize procurement	• Drugs purchased through several different flexible funds.<br>• List of required inputs created and procured by hospital administration. (e.g., for autoclave process - number of delivery trays, instruments, linen, etc.).<br>• Shift-in-charge and labour-room-in-charge staff used registers to support handover-takeover and stock maintenance processes.

2. Data Systems to Support Improvement

2.1 Build Reliable Systems for Data Collection

Change Concept	Change Idea	Changes Implemented
Standardise data collection	Complete a daily and monthly summary and review of records	• Used defined format for daily entry of key labour room data.<br>• All admissions included in LR register.<br>• LAMA/Abscond/Discharge/Triage added to the register.<br>• Built awareness about data collection.<br>• Clearly defined roles and responsibilities and established accountability.<br>• Assurance given by leadership for no blame culture.<br>• Frontline staff led work.<br>• Created a schedule for weekly register and data review.<br>• Created line listing of all the registers.

2.2 Strengthen Data Quality

Change Concept	Change Idea	Implementation
Create transparency in data collection and use	Track and address data, defects, and results for patient-centred healthcare	• QI teams tracked key maternal and new-born health data indicators.<br>• QI teams ensured both daily and monthly compilation of data, transparent reporting, and public display of achievement of the hospital.<br>• Regular updates on QI work were given to district and state health leadership for their support and ownership.

2.3 Use Data for Improvement

Change Concept	Change Idea	Implementation
Use available data appropriately for continuous improvement	Use data in QI meetings	• Data shared in weekly clinical review meetings.<br>• Garnered leadership attention, e.g., data displayed in district program management (DPM) office.
	Incorporate storytelling	• Stories were shared at QI meetings and weekly clinical reviews.
	Use visual display boards	• Created run charts with ongoing QI project details and relevant data, e.g., percentage compliance with real time partograph filling, maternal complication identification in labour room etc. Used for handover and takeover of women with pregnancy related complications.

	Use data for decision making	• QI team openly discussed clinical errors during intrapartum and postpartum care and tried to rectify any deviance from protocolized management.<br>• Engaged state and district leadership (i.e., district magistrates) in data review related to QI aims - strengthened accountability mechanisms.

3. Quality Management System

The essential components of a quality management system include engaged and accountable leadership, frontline improvement teams supported to use QI, and an established learning system across district hospitals. The following describes how each of these components were tested among the 10 district hospitals.

3.1 Build Leadership Accountability

Change Concept	Change Idea	Changes Implemented
Create vision and build will	Regularly review and follow-up QI work	• Leadership meetings held with Civil Surgeons and District Program Managers to generate willingness and sense of accountability to regularly support and review QI work in district hospitals.

3.2 Create and Nurture Frontline teams

Change Concept	Change Idea	Changes Implemented
Change the culture to support improvement	Acknowledge and support improvement efforts	• Felicitation of champions by awarding *Best Performer of the Month* from hospital leadership - generating joy, pride, and extrinsic motivation at workplace.<br>• Willingness of hospital leadership to optimize utilization of available human resources (via transparent duty rosters, procurement of instrument, supplies and consumables, and proactive resolution of issues), producing an enabling work environment and intrinsic motivation in staff for patient-centric care.

3.3 Build a Cross-district Learning System

Change Concept	Change Idea	Changes Implemented
Build QI capability	Build and support	• In QI meetings, staff participated in transparent discussions where they looked at key performance indicators such as

	functional QI teams	maternal complication identification and management, emergency c-section, blood transfusion, LAMA, absconds, referrals, deaths, etc.<br>• Publicly displayed outcomes and key performance indicators - generating higher degree of provider accountability.

4. Improve Quality of Clinical Care

4.1 Effective Triage of Pregnant Women on Admission

Change Concept	Change Idea	Changes Implemented
Redesign the triage process	Ensure resource availability	• Ensured availability of case sheets.
	Implement colour coding triage system	• Triage was done on admission.<br>• Used colour coding of high-risk cases.<br>• Complications recorded in bold letters to catch attention.
	Sensitize frontline staff on common issues	• Filled real time partographs.<br>• Emphasised importance of effective case handover.<br>• Clinical discussion held weekly or fortnightly by team.<br>• Collaboration and learning were done among peer hospitals.<br>• Application of QI principles the DH teams helped move from a culture of inspection and audit, to engagement and honest discussions around identification of gaps and possible solutions.

4.2 Increase Identification of Pregnancy-related Complications

Change Concept	Change Idea	Changes Implemented
Create urgency	Provide appropriate education on maternal complications	• Sensitized nurses on pregnancy complications.<br>• Used triage system.<br>• Shared real stories and data of harm

	through different mechanisms	• Shared QI learning on maternal complication identification in hospital and district meetings.<br>• Incorporated AMANAT clinical training programs in QI work.<br>• Aligned Ministry of Health and Family Welfare LaQshya program indicators with QI work to raise leadership attention.
Ensure accurate and reliable monitoring	Improve quality of documentation	• Increased use of safe birth checklist.<br>• Emphasized real time partograph monitoring in labour room and recorded compliance.<br>• Emphasized taking history on admission.<br>• Performed labour room register audit<br>• Nurse-in-charge reviewed case sheets to identify complication cases not reported.
	Develop measurement framework to guide improvement	• Operational definitions defined with inclusion and exclusion criteria<br>• Outcome, process, and balancing measures defined, and measurement plan was developed.<br>• Data collected and analysed by frontline staff<br>• QI visual boards displayed in the facilities and regularly updated on monthly basis, along with other improvement tools. Included daily cases as well.<br>• Developed process for daily tally and monthly summary of identified maternal complications. Complication register maintained.
Maintain continuity of necessary supplies	Ensure timely supply of necessary equipment	• Purchased instruments locally.<br>• Usage data sent to District Magistrate (DM) and other leaders.
		•

4.3 Increase Indicated C-Sections

Change Concept	Change Idea	Changes Implemented
Increase accountability	Form a c-section response team	• Team consisted of surgeons, duty doctors, nurses, DS, Hospital Manager (HM), District Technical Officers (DTOF).<br>• Labour room nurse communicated to operating surgeon all complications requiring c- section via phone and recorded it in

		call book register.<br>• General surgeon engaged to perform c-section besides obstetrician and EmOC doctors
	Involve leadership to remove barriers and facilitate changes	• Used existing platforms such as District Quality Improvement Team (DQIT) and District Quality Assurance Committee (DQAC) to engage with leadership on a regular basis – critical in removing barriers.<br>• Set regular, recurring meetings.<br>• Encouraged proper documentation of c-section and indications.
Redesign process to identify and manage maternal complications resulting in c-section	Sensitize nurses on indications for c-section	• Dedicated space for triage assessment with required equipment.<br>• Used triage form with colour coding (red, yellow, green) as per risk identified.<br>• Trained nurses on use of triage form and the colour coding categories.
	Redesign OT for hassle-free c-section services	• Clarified staff roles.<br>• Defined purpose of each physical space.<br>• Developed standardised processes.<br>• *See Additional ideas for more detail*
	Conduct referrals with two-way timely communication between Primary Health Centre (PHC) and DH	• Used WhatsApp group and landline telephone to communicate referral of maternal and newborn complications.<br>• Created 24x7 Referral Support WhatsApp group for all district staff working in labor rooms.<br>• Patient feedback collected using audio visual interviews. These interviews were shown to doctors to show importance of the change.
	Ensure post-operative monitoring compliance	• Used standardised policy with defined responsibility for post-operative monitoring and clinical training.
Redesign instrument sterilization and procurement process	Designate area for CSSD	• Designed room for CSSD service.<br>• Hospital management designated space for cleaning linen, disinfection, wrapping sets, and autoclaving.

	Define roles and responsibilities	• Staff was designated for CSSD service and roles and responsibility were assigned.<br>• *See Additional ideas for more detail on instrument sterilization roles and responsibilities.*
	Regularly monitor compliance	• Placed autoclave register and daily checklist for sterile set stock in each closed/opened drum.
	Standardise procurement of required instruments	• *See Additional ideas 1 for more detail regarding instrument procurement.*
Increase availability of key services and resources	Increase doctor availability for c-sections	• More doctors were posted in labour room with leadership intervention.<br>• More gynaecologists were engaged and involving in the QI meetings.
	Ensure emergency laboratory tests are available 24/7	• Laboratory technician roster prepared and implemented with the help of leadership.
Redesign instrument sterilization and procurement process	Designate area for CSSD	• Designed room for CSSD service.<br>• Hospital management designated space for cleaning linen, disinfection, wrapping sets, and autoclaving.
	Define roles and responsibilities	• Staff was designated for CSSD service and roles and responsibility were assigned.<br>• *See Additional ideas for more detail on instrument sterilization roles and responsibilities.*
	Regularly monitor compliance	• Placed autoclave register and daily checklist for sterile set stock in each closed/opened drum.
	Standardise procurement of required instruments	• *See Appendix 1 for more detail regarding instrument procurement.*

4.4. Increase Blood Transfusion for Pregnancy Complications (if indicated)

Change Concept	Change Idea	Changes Implemented

Redesign the blood transfusion acquisition process	Communicate need for blood in a timely way	• Labour room nurse called and sent requisition form to Blood Bank LT and BB MOIC for all complications requiring c-section.<br>• QRT formed WhatsApp group for communicating blood need.
	Leadership authorizes emergency cases	• Emergency department (ED) letter authorized provision of blood without replacement during emergency cases.
	Use data collection, monitoring, and dissemination	• Reviewed blood transfusion and c-section data during either the weekly QI meeting or monthly DM meeting.<br>• Documented and reviewed any c-section complication attributed to blood arrangement.
	Increase availability of blood	• Held voluntary blood donation camps.<br>• Created 24/7 blood bank technician roster.

4.5 Increase Referrals from Primary Health Centres to District Hospital

Change Concept	Change Idea	Changes Implemented
Identify and refer cases in timely manner	Provide training to staff on clinical knowledge and improvement skills	• Referral slip given to each referral case. All given medication was noted in referral slip.<br>• Ambulance notified of proper drop off location.<br>• ANMs trained where and how to refer cases.<br>• Workshops organized by the state to strengthen ANMs, ASHAs and other related providers.<br>• Facility assigned individual(s) to receive patients and communicate with doctors.
	Establish reliable communication across clinical teams for refereed patient	• WhatsApp group created to inform about timely referrals, data assessment, feedback, etc.<br>• Telephone system established in labour rooms of each facility.<br>• Telephone number of referred facilities displayed labour room to increase follow-up.<br>• Referred out cases followed-up by officials.<br>• Used closed loop communication method; Once patient reached District Hospital, the Primary Health Center was informed.<br>• Ambulance service number made available at labour room and LR ANMs.

	Improve data collection and reporting	• Referral out register kept in each facility for documentation.<br>• Referral out register included mobile number of ASHA and patient.<br>• Nurses trained to collect data.<br>• Data collected and analysed by frontline staff.

Additional Change Idea Details

Change Idea: Clarify Roles and Responsibilities for Optimal Use of Resources

Changes Implemented

Nurses

- **Nursing roster**: with leadership support, 16 motivated nurses were identified and placed in roster. This required discussion with Medical Superintendent and Civil Surgeon.
- **Defined Role and Responsibility**: Four teams (A, B, C, D Teams) consisting of the following individuals were made in nursing roster:
  - 1 shift-in-charge
  - 2 nurses
  - 1 sweeper

  Each team was on duty for two continuous days followed by change in duty. The shift-in-charge was called for QI meetings, and a hospital manager communicated all instructions.
- One nurse was made responsible for triage and ANC ward, and the nursing station was placed nearby. Two nurses made responsible for conducting deliveries in delivery room.

Doctors

- District Magistrate, Civil Surgeon, and DS worked together to implement 24*7 roster for doctors.
- Improved doctors' room and provided amenities to make it comfortable for doctors to stay overnight in facility.
- Local role models built will for roster compliance.

Mamtas

- Created MAMTAs roster, and a team of MAMTAs was assigned to each shift.
- Within each team, clear roles and responsibilities were assigned (e.g., registration counter, antenatal wards, etc.).
- Trained MAMTAs on assigned responsibility (e.g., registration of new admissions, counselling at time of admission, postnatal counselling, etc.).

**OT Technician**<br>• Contractually hired OT staff by district hospital.<br>• Posted OT technician from PHC to district hospital.<br>• Created roster for OT staff.<br>• Local training provided on role and responsibility.<br>• OT technician was assigned the additional responsibilities of post c-section wound dressing, register maintenance for surveillance of surgical site infections, and autoclaving OT instruments and linen.
**Sweeper**<br>• Sweeper roster created with one sweeper assigned to team of nurses in every shift. The shift-in-charge was then made responsible for supervising the cleaning process and ensuring cleanliness is maintained.
**Lab Technician**<br>• Roster for lab technicians created to ensure 24*7 emergency lab tests.
**Blood Bank Technician**<br>• Roster for blood bank technicians created to ensure 24*7 availability of blood bank staff to crossmatch and provide blood.
**Dedicated/assigned person for centralized autoclave**<br>• Contractually hired an individual through local DH funds that was trained to autoclave OT and delivery room instruments.<br>• OT technician assigned responsibility of autoclaving OT instruments and linen.

Changes Implemented
**Clarified staff roles**<br>• Nurses were made OT-in-charge and LR-in-charge respectively.<br>• 24x7 duty roster was prepared for doctors, GNM and ANM, and grade-IV staff.<br>• MAMTAs were assigned the task of cleaning instruments and equipment, preparing surgical pads, and counselling mothers in postoperative surgical ward and maternity ward.
**Defined purpose of each physical space**<br>• Two separate OTs were developed for c-section and tubal ligation. The old labour room was reorganized into postoperative surgical ward. OT and LR were separated from each other.<br>• OT divided into zones (i.e., Sterile Zone, Protective Zone, Clean Zone and Dirty Zone).
**Developed standardised processes**<br>• Developed standard operating and handoff procedure in local language (Hindi).<br>• Standardised location of notes in case sheet<br>• All the nurses were trained by the head nurse.

Change Idea: Redesign Operation Theatre for Hassle-free Caesarean Section Service

- A case sheet audit tool was prepared for the Hospital Manager to check the process compliance on a monthly cadence.

Change Idea: Define Instrument Sterilization Roles and Responsibilities

Changes Implemented

- Tasks included:
    - Disinfection
    - Cleaning
    - Wrapping
    - Transport to CSSD room and back
    - Checking at LR
    - Monitoring autoclaving
    - Filling-up autoclave register
    - Moving autoclave register to and from CSSD
- Grade-IV staff and MAMTAs
    - Disinfected contaminated instrument in freshly prepared 0.5% bleaching powder solution after every delivery, cleaned, and dried instruments. All the contaminated instruments are disinfected in the labour room.
        - The scissors are separated at this point and are sterilized by using 2%-Glutaraldehyde.
    - Wrapped sets (except scissors) in designated space. The packed instruments are transferred to the drum and sent for autoclaving along with autoclave register. LR nurse enters number of sets sent in each drum for autoclave. The drums and associated register are sent along with MAMTA to the sterilization room located in the OT complex.
- Every morning, the previous day’s instrument sets are sent for sterilization. This happens only once in a day. An individual was hired for CSSD by using Rogi Kalyan Samities (RKS) funds.
- After sterilization, the drums and register are brought back to LR. The time of sterilization was noted, and a singular tape is pasted in the register.
- The LR nurse maintains a shift wise record of number of drums available, open/ closed and sets available in open/closed drums.
- Sterile packs are prepared and kept on the right side of the table. Each one is opened during the delivery, used, transferred into the buckets with hypochlorite solution, cleaned, washed, and dried by Grade-IV staff and MAMTAs. Drying was done on the crash cart. Packed instruments are transferred to the drums, labelled, and kept till it gets transferred to the CSSD room.
- Sterilization of Scissors: Three separate glutaraldehyde (Cidex) trays (i.e., A, B, C) and a saline tray were kept for sharp instruments used in morning, evening, and night shifts respectively. For example, the scissors used in the morning shift are kept in the morning shift Cidex tray-A, scissors used in evening shift are kept in evening tray-B, and scissors used in the night shift were kept in night tray-C respectively. This change idea clicked.

- These instruments were kept in 2%-glutaraldehyde for a maximum time of 16 hours before being taken out for use on next day, ensuring a prescribed holding time of 10-24 hours with 2%-glutaraldehyde solution. Sharp instruments like plain scissor, cord cutting scissors and Episiotomy scissors are kept in 2%-glutaraldehyde.

Change Idea: Standardise Instrument Procurement Process

Changes Implemented

- Following logistics, instrument and equipment were procured
  - Labour Room Delivery Sets were increased from 14 to 26
  - Drums were increased from 2 to 6
  - Wrapping cloths were increased from 50 to 100
  - Drums were labelled by numbers
- Number of supplies:
  - 30 delivery sets (each delivery set contains - 2 artery forceps,1 sim's speculum, 2 sponge holding forceps, kidney tray, 1 cord cutting scissor)
  - 8 episiotomy sets (each episiotomy set contains – 1 allis forceps, 1 thumb forceps, 1 needle holder, 2 artery forceps,1 sim's speculum, 2 sponge holding forceps, kidney tray, cord cutting scissor,1 episiotomy scissor)
  - 45 delivery and episiotomy sets were also procured for back-up and kept in store
  - 10 autoclave drums for instruments, sanitary pads, and other consumables
  - 80 cord cutting scissors
  - Bleaching powder, Cidex (2%-Glutaraldehyde)
  - Washing machine for washing of clothes
- Three-trays system (A, B, C – for morning, evening and night shift respectively) were implemented for chemical sterilization of sharp instruments.
